# Metabolic Responses to Waterlogging Differ between Roots and Shoots and Reflect Phloem Transport Alteration in *Medicago truncatula*

**DOI:** 10.3390/plants9101373

**Published:** 2020-10-15

**Authors:** Jérémy Lothier, Houssein Diab, Caroline Cukier, Anis M. Limami, Guillaume Tcherkez

**Affiliations:** 1 Seedling Metabolism and Stress, Université d’Angers, Agrocampus Ouest, INRAE, UMR IRHS, SFR QuaSaV, 49071 Beaucouzé, France; drhd85@hotmail.com (H.D.); caroline.cukier@univ-angers.fr (C.C.); anis.limami@univ-angers.fr (A.M.L.); 2 Research School of Biology, ANU Joint College of Science, Australian National University, Canberra, ACT 2601, Australia

**Keywords:** waterlogging, hypoxia, metabolomics, phloem

## Abstract

Root oxygen deficiency that is induced by flooding (waterlogging) is a common situation in many agricultural areas, causing considerable loss in yield and productivity. Physiological and metabolic acclimation to hypoxia has mostly been studied on roots or whole seedlings under full submergence. The metabolic difference between shoots and roots during waterlogging, and how roots and shoots communicate in such a situation is much less known. In particular, the metabolic acclimation in shoots and how this, in turn, impacts on roots metabolism is not well documented. Here, we monitored changes in the metabolome of roots and shoots of barrel clover (*Medicago truncatula*), growth, and gas-exchange, and analyzed phloem sap exudate composition. Roots exhibited a typical response to hypoxia, such as γ-aminobutyrate and alanine accumulation, as well as a strong decline in raffinose, sucrose, hexoses, and pentoses. Leaves exhibited a strong increase in starch, sugars, sugar derivatives, and phenolics (tyrosine, tryptophan, phenylalanine, benzoate, ferulate), suggesting an inhibition of sugar export and their alternative utilization by aromatic compounds production via pentose phosphates and phospho*enol*pyruvate. Accordingly, there was an enrichment in sugars and a decline in organic acids in phloem sap exudates under waterlogging. Mass-balance calculations further suggest an increased imbalance between loading by shoots and unloading by roots under waterlogging. Taken as a whole, our results are consistent with the inhibition of sugar import by waterlogged roots, leading to an increase in phloem sugar pool, which, in turn, exert negative feedback on sugar metabolism and utilization in shoots.

## 1. Introduction

Flooding is currently one of the most severe factors that reduces crop productivity [1,2]. It is believed that at least 10% of arable fields can be affected by flooding (FAOSTAT, www.fao.org). As such, it concerns many cultivated species, including major crops, like wheat, and alternative agricultural practices have to be found to overcome this problem (reviewed in Manik et al., 2019 [3]). Flooding leads to occasional or prolonged root submergence (waterlogging). O_2_ deprivation is the most important biochemical factor during waterlogging [4]: in fact, when air spaces that are normally present in soil are filled with water, root environment becomes hypoxic due to O_2_ consumption by respiring roots (and microorganisms). The well-known effects of hypoxia relate to energy limitation (i.e. reduction in respiratory ATP production due to O_2_ shortage) and cytoplasm acidification due to a decline in plasma membrane H^+^-ATPase activity as well as organic acid generation by metabolism [5]. Although prolonged hypoxia strongly impacts on growth and survival [6], occasional hypoxia can be accommodated via the expression of so-called “core hypoxia-responsive genes” (reviewed in Hsu & Shih, 2013, [7]). Such a genetic response leads to a re-orchestration of root primary metabolism, including (*i*) the induction of ethanolic fermentation (e.g. induction of alcohol dehydrogenase and pyruvate decarboxylase, and in soybean, an increase in enolase and phosphoglycerate kinase [8]), (*ii*) an increase in sucrose synthase to metabolize sugars, and (*iii*) the accumulation of metabolites from alternative pathways e.g. γ-aminobutyrate (GABA) and alanine [9]. Alanine accumulation is a hypoxic biomarker metabolite (reviewed in Planchet et al., 2017, [10]) and, accordingly, a gene encoding alanine aminotransferase has been found amongst core hypoxia-response genes [11].

Up to now, most of the studies regarding hypoxia utilize roots or whole seedlings kept in darkness in a N_2_ atmosphere or under submergence [12,13,14,15,16,17]. Although valuable in understanding root metabolism during waterlogging, these studies provide limited information to delineate metabolic responses at the whole-plant level and potential metabolic differences in other organs. In effect, roots and shoots do not exhibit the same response to hypoxia [18,19,20,21,22,23]. Recently, Mustroph et al., 2014 [24] addressed this question via a meta-analysis of published data that were obtained in Arabidopsis (*Arabidopsis thaliana)*. They found that roots respond more strongly and typically produce GABA, alanine, and lactate, while shoots seem to have less hypoxic symptoms (but accumulate alanine), probably due to O_2_ generation by photosynthesis.

In adult plants (rather than seedlings) and in other species, organ-specific metabolic effects of waterlogging are much less known. An accumulation of starch in leaves of waterlogged plants is generally observed (reviewed in Irfan et al., 2010 [25]). Recently, detailed metabolomics analyses have been carried out in sunflower (*Helianthus annuus*) and oil palm (*Elaeis guineensis*) [26,27]. In both species, waterlogging leads to an increase in alanine, GABA, and polyols (glycerol, inositol, ononitol), changes in organic acids (citrate, aconitate, maleate), and a decrease in many amino acids, showing the limitation of catabolism (respiration) and the inhibition of the tricarboxylic acid pathway, which, in turn, impacts on amino acid homeostasis. In leaves, waterlogging leads to a strong increase in sugars (hexoses and polyols derived therefrom) and a decline in several amino acids (sometimes including alanine), suggesting an inhibition of sugar export and N assimilation [26]. In addition, there is a decline in nitrate content in leaves, much less pronounced in roots, suggesting an inhibition of xylem nitrate transport and in fact, computations based on ^15^N natural abundance have shown a strong effect of waterlogging on nitrate circulation [28]. Additionally, in cotton, waterlogging causes a decline in the expression of nitrite reductase and an increase in nitrite concentration in leaves [29]. In nodulated soybean (*Glycine max*), waterlogging inhibits the transport of ureides and glutamine to the shoot via the xylem [30,31]. Taken as a whole, waterlogging has important consequences on leaf metabolism, including N assimilation, and part of this effect is likely caused by a perturbation of metabolite exchange via phloem (and xylem) sap. In effect, in castor bean (*Ricinus communis*, a species that allows facile phloem sap sampling) under waterlogging, phloem sap flow, and conducting area have been found to decrease while phloem sap sugar concentration slightly increases [32]. Also, in French bean (*Phaseolus vulgaris*) cultivated hydroponically, ^14^C translocation to roots after the isotopic labelling of leaves with ^14^C-sucrose is approximately 50% lower when roots are under anoxic conditions [33]. Similarly, when a non-metabolizable radioactive glucose analogue (^14^C-1-deoxyglucose) is fed to shoots of maize seedlings, the translocation of radioactivity does not reach root tips when the root is kept under anoxic conditions [34].

Nevertheless, in our present knowledge of systemic effects of waterlogging, uncertainty remains as to (*i*) how leaf and root metabolome are coordinated, (*ii*) whether both phloem sap composition and transport rate are affected, and, if so, (*iii*) whether phloem loading or unloading (or both) is primarily affected. Here, we used non-nodulated barrel clover (*Medicago truncatula*) plants and carried out a systematic metabolic analysis using GC-MS profiling in roots, leaves, and also phloem sap exudates at different time points after the onset of waterlogging. We also carried out gas-exchange measurements and elemental content (C, N) determination. We used barrel clover as a model species because of its importance as a cultivated forage crop in the Mediterranean region and Australia, where waterlogging stress periodically occurs due to seasonal heavy rain events. Additionally, we focused on non-nodulated plants, since the establishment of N_2_-fixing symbiosis considerably modifies nitrogen metabolism (in favor of ureides, amides, etc.) and the impact of waterlogging on *Rhizobium* would have had to be taken into account. Additionally, this will facilitate the comparison with plants devoid of N_2_-fixation in the discussion. We found metabolic changes that are consistent with an inhibition of sugar import (unloading) by waterlogged roots, leading to an increase in the phloem sugar pool, which, in turn, exerts a feedback on sugar export and metabolism in leaves.

## 2. Results

### 2.1. Photosynthesis and C:N Composition

**Figure 1:**
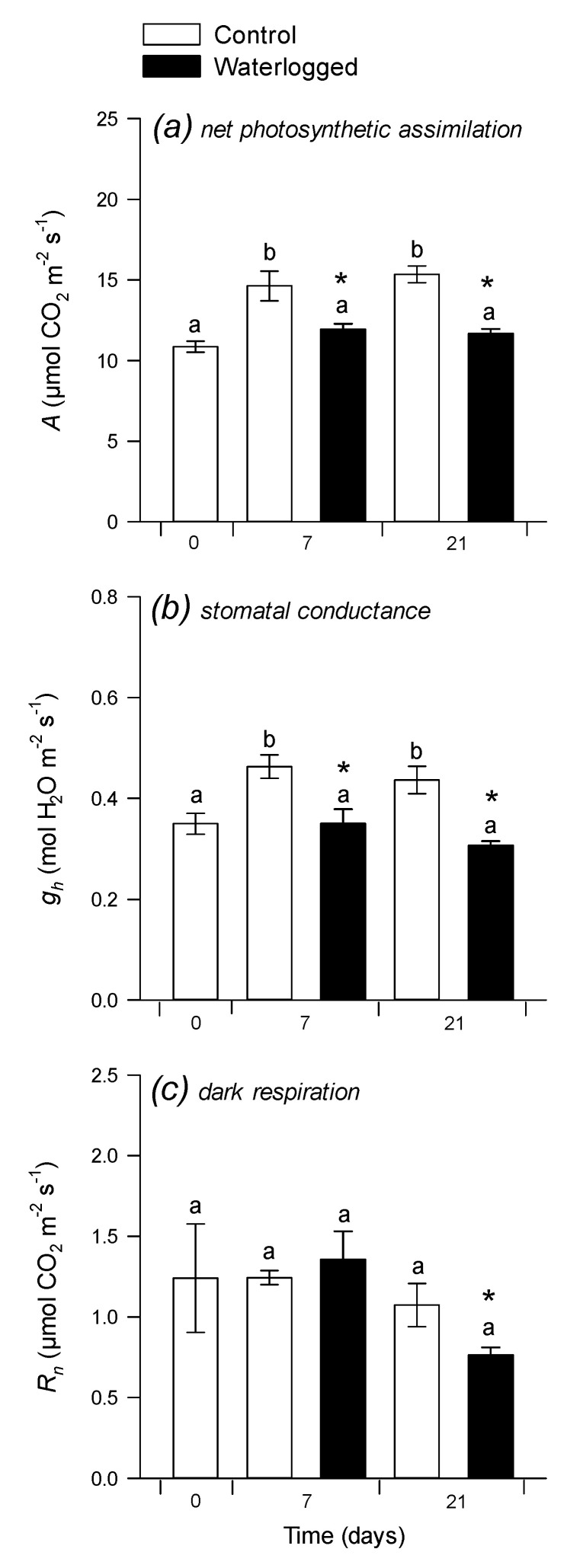
Leaf gas-exchange parameters: net photosynthetic CO_2_ assimilation. (**a**), stomatal conductance (**b**) and dark respiration (**c**) 7 and 21 days after the beginning of waterlogging. The zero-time point was just before the onset of waterlogging. Non-waterlogged (control) plants are shown with open bars, waterlogged plants with black bars. Gas exchange was carried out under 380 µmol mol^−1^ CO_2_ and 21% O_2_ at 400 µmol m^−2^ s^−1^ PPFD. Data are means ± SE (*n* = 4). Letters stand for significantly different statistical classes (one-way ANOVA, *p* < 0.05 with post-hoc Tukey test). In (**c**), the asterisk stands for significance (*p* < 0.05, Welch test) for pair-wise comparison waterlogging vs. control.

Leaf photosynthesis (net CO_2_ assimilation) was measured under standard conditions (380 µmol mol^−1^ CO_2_, 21% O_2_). As expected, there was a significant negative effect of waterlogging on photosynthesis (Figure 1a), due to lower stomatal conductance (Figure 1b). Interestingly, this effect was not more pronounced after 21 d (as compared to 7 d) suggesting that leaf photosynthesis partly acclimated to waterlogging conditions. By contrast, CO_2_ evolution in darkness was minimally affected after 7 d and was significantly lower under waterlogging after 21 d (Figure 1c). The overall result of lower carbon assimilation was a lower dry weight (DW) in roots after 7 d and in both roots and shoots after 21 d (Figure S1a,b). Interestingly, the relative water content was higher by 1–2% under waterlogging (Figure S1c,d), which partly compensated for the effect of waterlogging on DW in fresh matter production. 

**Figure 2:**
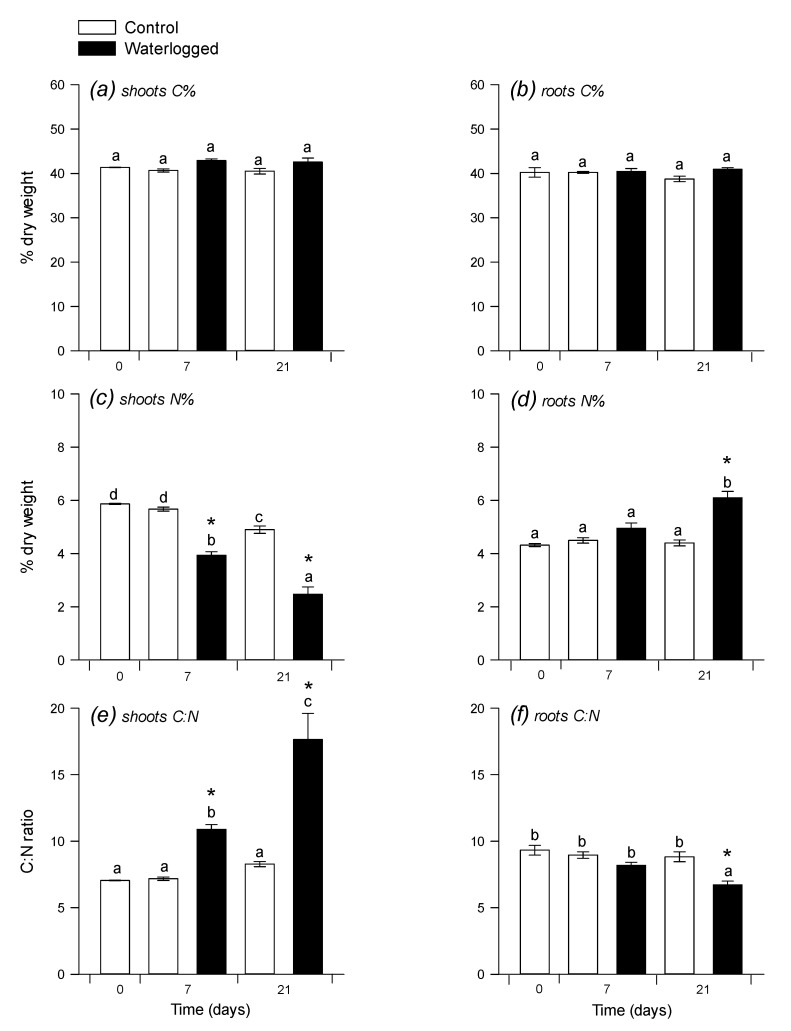
Carbon and nitrogen elemental content. C concentration in shoots (**a**) and roots (**b**) N concentration in shoots (**c**) and roots (**d**) and C:N ratio in shoots (**e**) and roots (**f**) Same legend as in Figure 1. Data are means ± SE (*n* = 3).

**Figure 3:**
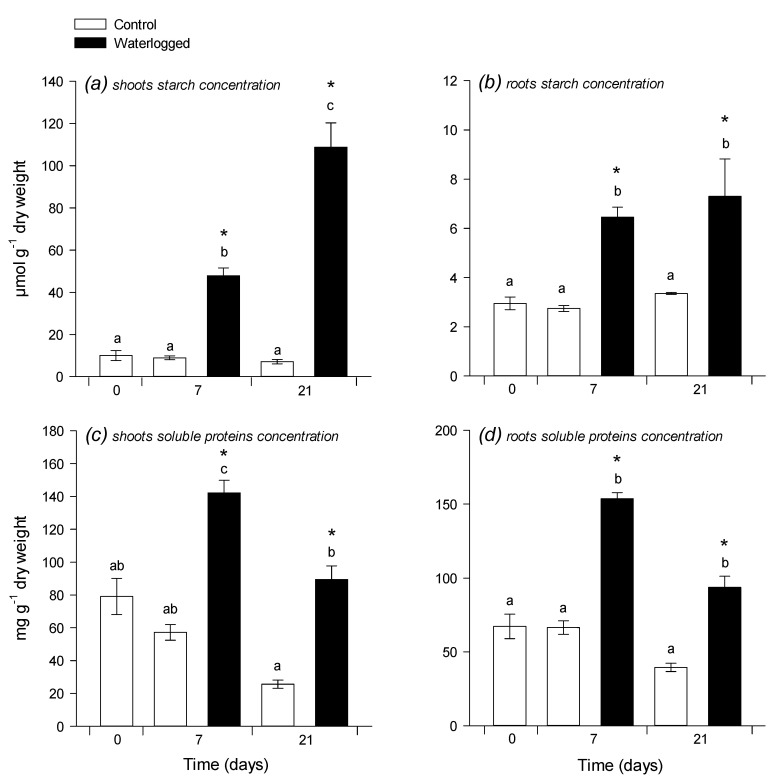
Starch and soluble protein concentrations. Starch content (in µmol hexose equivalents g^−1^ DW) in shoots (**a**) and roots (**b**) and soluble proteins concentration (in mg g^−1^ DW) in shoots (**c**) and roots (**d**) 7 and 21 days after the beginning of waterlogging. Same legend as in Figure 1. Data are means ± SE (*n* = 3).

There was no significant change in the carbon elemental content (%C), whereas nitrogen elemental content decreased in shoots and increased (at 21 d) in roots under waterlogging (Figure 2a–d). As a result, after 21 d, the C:N ratio increased a lot in shoots (nearly two-fold increase) and decreased slightly (by about 25%) in roots (Figure 2e,f). Accordingly, there was a four- to 10-fold increase in starch content and 2-fold increase in protein content (expressed in mg g^−1^ DW) in shoots under waterlogging (Figure 3a–c), while, in roots, both starch and proteins increased 2-fold (Figure 3b–d).

### 2.2. Metabolomics Pattern in Leaves and Roots

**Figure 4:**
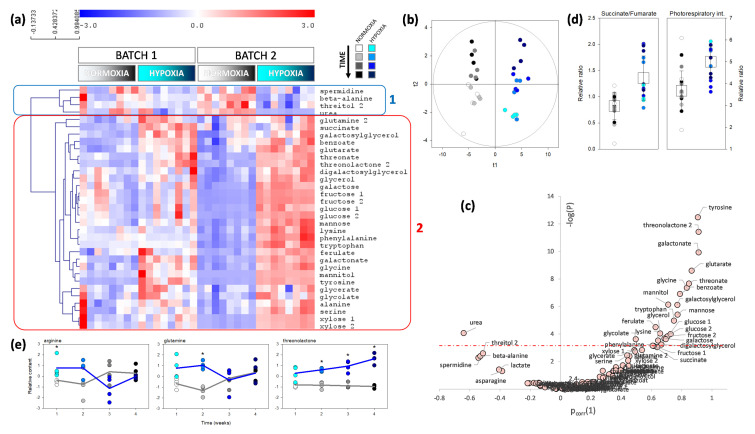
Metabolomics pattern of leaves when roots of the same plant are under control (normoxia) or waterlogging (hypoxia) (grey and blue shades, respectively). (**a**) Heat map showing significant metabolites (*p* < 0.01; two-way ANOVA) with a hierarchical clustering on left (Pearson correlation). The two main groups are framed and numbered (group 1 framed in blue: metabolites decreasing under hypoxia; group 2 framed in red: metabolites increasing under hypoxia). (**b**) Score plot of the multivariate analysis by O2PLS demonstrating the very good sample discrimination. (**c**) Volcano plot showing best discriminating metabolites (waterlogging vs. control) with the *p*-value (*y*-axis) and the loading in the O2PLS (*x*-axis). The horizontal dash-dotted line represents the Bonferroni threshold (0.0005). The two best discriminating features under waterlogging are an increase in tyrosine and a decrease in urea. (**d**) Relative metabolic ratio succinate-to-fumarate (left) and percentage (%) of photorespiratory intermediates (serine + glycine + glycolate + glycerate) in total metabolites (right). For both, the difference between waterlogging and control is significant (*p* < 0.01, Welch). (**e**) Metabolites that have a significant waterlogging × time interaction effect (arginine, glutamine, threonolactone). Conditions under which the difference between control and waterlogging is significant is labelled with an asterisk (*).

Because both C assimilation and N content were affected by waterlogging, we carried out an in-depth analysis with metabolic profiling to identify metabolites of primary C and N metabolism influenced by waterlogging. Metabolomics analyses were carried out on leaves and roots that were collected over four weeks, using GC-MS. This allowed for us to identify and quantify 123 analytes. The effect of waterlogging was analyzed using both univariate (two-way ANOVA) and multivariate (O2PLS) analyses, where two factors were considered (time and waterlogging). In leaves, 33 analytes were found to be significantly affected (*p* < 0.01) by waterlogging, and formed two clusters (labelled 1 and 2 in Figure 4). The first cluster comprised nitrogenous compounds (urea, spermidine, and β-alanine) as well as threitol, which were less abundant under waterlogging. The second cluster was made of metabolites that were more abundant under waterlogging, mostly sugars (fructose, galactose, glucose, mannose, xylose), organic acids (such as succinate, glutarate), aromatics (tyrosine, phenylalanine, tryptophan, ferulate), and some amino acids (glycine, alanine, glutamine, lysine). The multivariate analysis yielded a predictive (R^2^ = 0.88) and robust (Q^2^ = 0.66) model that was highly significant (*P*_CV-ANOVA_ = 2.4∙10^−9^) and separated control and waterlogged conditions easily (Figure 4b). When univariate and multivariate analyses are combined into a volcano plot, the best biomarkers of waterlogging appeared to be tyrosine, threonolactone and galactonate (increased), and urea (decreased) (Figure 4c). Interestingly, amongst the ratios of organic acids, the succinate-to-fumarate ratio was significantly higher under waterlogging (Figure 4d), which suggested a specific downregulation of succinate oxidation to fumarate in the Krebs cycle and/or fumarate generation by polyamine synthesis. Additionally, there was a significant increase in photorespiratory intermediates (sum of serine, glycine, glycolate, and glycerate) under waterlogging, which suggested a higher photorespiration rate. This would agree with the lower stomatal conductance that was found by gas exchange (Figure 1b). Three metabolites had a significant interaction (time × waterlogging) effect (Figure 4e): arginine and glutamine (which were significantly higher only at the beginning of waterlogging), and threonolactone (which increased under waterlogging, while it remained constant under control conditions).

**Figure 5:**
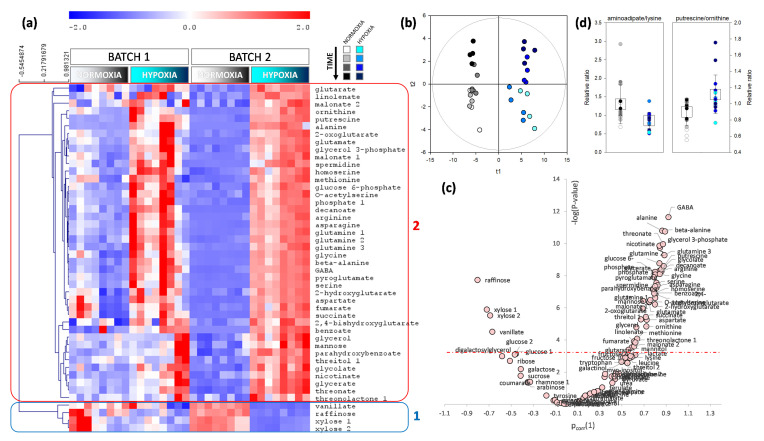
Metabolomics pattern of roots under control (normoxia) or waterlogging (hypoxia) conditions (grey and blue shades, respectively). (**a**) Heat map showing significant metabolites (*p* < 0.0005, i.e. Bonferroni threshold; two-way ANOVA) with a hierarchical clustering on left (Pearson correlation). The two main groups are framed and numbered (group 1 framed in blue: metabolites decreasing under waterlogging; group 2 framed in red: metabolites increasing under waterlogging). (**b**) Score plot of the multivariate analysis by O2PLS demonstrating the very good sample discrimination. (**c**) Volcano plot showing best discriminating metabolites (control vs. waterlogging) with the *p*-value (*y*-axis) and the loading in the O2PLS (*x*-axis). The horizontal dash-dotted line represents the Bonferroni threshold (0.0005). The two best discriminating features under waterlogging are an increase in γ-aminobutyrate (GABA) and a decrease in raffinose. (**d**) Relative metabolic ratios: aminoadipate-to-lysine (left) and putrescine-to-ornithine (right). For both, the difference between waterlogging and control conditions is significant (*p* < 0.01, Welch).

In roots, metabolites that were significantly more abundant under waterlogging were mostly nitrogenous compounds, including alanine and GABA, as well as glutamine, putrescine, spermidine, and arginine (Figure 5a). Only three metabolites were found to be less abundant under waterlogging: xylose, raffinose, and vanillate. The multivariate analysis yielded a predictive (R^2^ = 0.88) and robust (Q^2^ = 0.69) model that was highly significant (*P*_CV-ANOVA_ = 5.6∙10^−8^) and separated control and waterlogged conditions easily (Figure 5b). When univariate and multivariate analyses were combined into a volcano plot, the best biomarkers of waterlogging appeared to be alanine, GABA and β-alanine (increased) and raffinose (decreased) (Figure 5c). Interestingly, the aminoadipate-to-lysine ratio was significantly lower under waterlogging (Figure 4d), which suggested a specific downregulation of lysine catabolism. By contrast, the putrescine-to-ornithine ratio was significantly higher, showing a higher commitment to polyamine synthesis (Figure 5d).

### 2.3. Phloem Sap Composition and Movement

Because photosynthesis was altered while leaf sugar content increased, we explore the possibility that sugar transport via phloem sap was altered by waterlogging. Shoot phloem sap exudates were collected in EDTA solution in darkness. We collected phloem sap from whole shoots, since it was not possible to collect enough sap from individual leaves. In addition, collecting phloem sap exudated by whole shoots is useful for performing mass-balance at the plant scale and phloem transfer calculations. The metabolic composition was determined using both HPLC (sugars) and GC-MS (other metabolites). It is important to keep in mind that the exudation technique allows for quantifying the amount of metabolites of interest in the sample and, thus, to convert to a rate in nanomoles shoot^−1^ h^−1^. However, this cannot be converted into a concentration in phloem sap since the determination of the exudated (very small) volume is not straightforward. Also, the collection of the xylem sap failed under our conditions, including using a pressurized system (Scholander chamber). 

**Figure 6:**
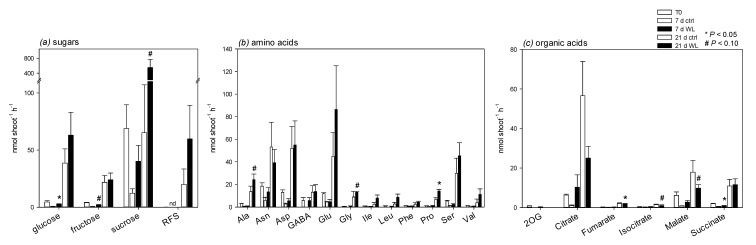
Composition of shoot phloem sap exudate (in EDTA solution) in darkness: sugars (**a**), amino acids (**b**) and organic acids (**c**). Results are expressed in exudation rate in nmol shoot^−1^ h^−1^. Absolute quantitation in moles was achieved by HLPC-conductimetry (sugars) or GC-MS (other metabolites). Abbreviations: RFS, raffinose-family sugars (raffinose, verbascose, stachyose); nd, not detected. Symbols (*, #) stand for statistical significance (Student-Welsh) between control (ctrl) and waterlogging (WL). Mean ± SE (*n* = 4).

Figure 6 shows the composition of phloem sap exudates. Unsurprisingly, sugars prevailed in phloem sap exudates. There was a general increase in sugar exudation under waterlogging (Figure 6a), although it was rather variable (sucrose was more abundant after 21 day under waterlogging, with a *p*-value of 0.07). There was little change in the amino acid composition (Figure 6b) of exudates, with the notable exception of glycine, proline, and alanine, which were more abundant under waterlogging after 21 day. Amongst organic acids, fumarate, isocitrate, and malate were less abundant under waterlogging after 21 day.

**Figure 7:**
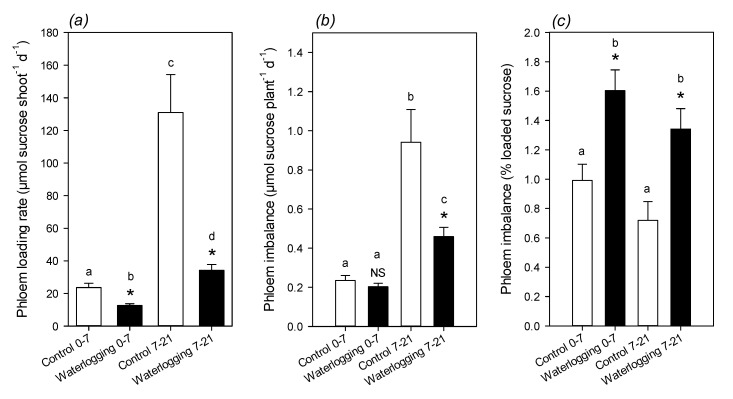
Calculated sucrose phloem loading rate and imbalance using mass-balance between shoots and roots: (**a**) average loading rate during for the two time windows considered (0 to 7 d, and 7 to 21 d) in µmol sucrose shoot^−1^ day^−1^; (**b**) imbalance (difference between loading by shoots and unloading by roots) in µmol sucrose plant^−1^ day^−1^; (**c**) imbalance expressed in percentage of loaded sucrose. Letters stand for significantly different classes in one-way ANOVA and asterisks stand for statistical significance (Welch test) between control and waterlogging. NS, non-significant. See Supplementary Material for calculation details.

The rate of phloem translocation was estimated while using mass-balance. Assuming that carbon transferred from shoots to roots was only made of sucrose, phloem loading and unloading rates were calculated, accounting for sucrose consumption for growth, starch synthesis, and respiration (Figure S2). Calculated average phloem loading rate was lower under waterlogging, in agreement with the lower assimilation rate and lower growth (Figure 7a). The difference between the loading by shoots and unloading by roots was always very small, in the order of 0.6 to 2% of loading; however, there was a clear increase in this difference under waterlogging (Figure 7b,c), showing that waterlogging inhibited phloem unloading by roots.

## 3. Discussion

### 3.1. Differential Effect of Waterlogging on Leaf and Root Metabolome

It is generally accepted that there are metabolic effects of waterlogging in leaves, despite the fact that waterlogging (as opposed to full submergence) does not lead to hypoxic conditions in leaves. A common symptom of waterlogging is leaf starch accumulation [25], suggesting a change in sugar export and/or in partitioning between sucrose and starch synthesis during photosynthesis. Here, we also found a strong increase in leaf starch content, which represented up to 1.8% of shoot DW (~1000 µmol hexose equivalents g^−1^ DW) (Figure 2). This effect is not caused by an increase in photosynthesis, since waterlogging rather induces a decline in CO_2_ assimilation (Figure 1). Hexoses (including galactose) and polyols (glycerol, mannitol) also both increased under waterlogging (Figure 4) and a similar effect has been found in other species [26,27]. The accumulation of hexoses under waterlogging is likely the result of the higher activity of sucrose synthase [9,10], which can regenerate UDP-glucose and, thus, in turn, feed galactose synthesis and metabolism (galactose, galactonate, threonate, and galactosylglycerol are significantly increased by waterlogging). Additionally, three metabolites of the polyamine pathway (urea, β-alanine, and spermidine) were less abundant, which suggested a downregulation of the entire pathway, which leads to a lower consumption and, in turn, a transient increase in arginine (Figure 4). This strong effect on polyamine metabolism has also been found in sunflower, including under K-deficient conditions, which normally induce putrescine accumulation [26]. The general increase in aromatics implies an increase in the consumption of their precursors, phospho*enol*pyruvate (PEP) and erythrose 4-phosphate, likely at the expense of both organic acid synthesis by PEP carboxylase and synthesis of erythrose derivatives, such as threitol (effectively decreased). The rationale of all of these metabolic changes probably relates to the stimulation of alternative pathways to consume hexoses via glycolysis, galactose metabolism, and the oxidative pentose phosphate pathway. 

There was a clear opposite effect of waterlogging on root metabolism, with less sugars (and one aromatic compound, vanillate), and an increase in hypoxic biomarkers (alanine, GABA) reflecting the recycling of pyruvate and 2-oxoglutarate when catabolism is inhibited by the lack of oxygen. In fact, waterlogging is accompanied by an inhibition of the Krebs cycle for two reasons: first, a limitation of NADH reoxidation due to low O_2_ concentration; second, hypoxia leads to an increase in nitric oxide (NO) production, which is a potent inhibitor of aconitase [35]. In effect, NO inhibits the second step (i.e., conversion of aconitate to isocitrate) of aconitase catalysis [36,37,38]. Additionally, there was an increase in many nitrogenous compounds, including β-alanine, putrescine, arginine, and the non-proteinogenic amino acid ornithine (Figure 5). This suggests that there was an increase in polyamines biosynthesis, which was perhaps associated with the urea cycle (urea was not significantly increased in roots under our conditions). Polyamines play a role in tolerance against abiotic stress, including hypoxia [39,40,41]. In addition, it has been proposed that the involvement of amino acid metabolism and polyamines under waterlogging could reflect an aspartate cycle [26], whereby arginine synthesis would represent a source of fumarate (citrulline + aspartate →→ arginine + fumarate), consumes reducing equivalents, and might be degraded to NO. Aspartate may then be reformed via malate and oxaloacetate (last steps of the Krebs cycle) or via the action of PEP carboxylase.

Interestingly, despite the general depletion in sugars and the high requirement to sustain substrate-level ATP synthesis (anaerobic glycolysis), waterlogged roots contained more starch (Figure 3), which suggested an inhibition of starch breakdown. This phenomenon is probably the result of the limitation in ATP generation and the utilization of glucose 6-phosphate for catabolism at the expense of phosphotransfer to glucose 1-phosphate and then starch non-reducing ends. 

### 3.2. Phloem Composition and Translocation under Waterlogging

The opposite situation that is associated with sugar content in leaves and roots suggests that the inhibition of sugar translocation via the phloem is a major driver of metabolic changes. Our analysis of exudate composition shows that sugar—in particular sucrose—was more abundant under waterlogging. This effect could have come from a higher sugar concentration in sap and/or a higher sap flow. It is rather unlikely that sap flow was larger, since waterlogging is generally associated with lower stem capacitance (hydraulic conductance) and low stomatal conductance (Figure 1), which, in principle, inhibits both xylem and phloem sap flow. In waterlogged castor bean plants, phloem sap flow (bleeding rate) has been found to be lower while sugar concentration was slightly higher [32]. In *Eucalyptus*, waterlogging leads to a decrease in sucrose, but a strong increase in raffinose in phloem sap [42]. Thus, it appears more likely that the sucrose concentration in phloem sap was higher under waterlogging, and this was caused by an inhibition of translocation. Our mass-balance calculation further supports this hypothesis, with a higher relative imbalance (loading rate minus unloading rate) under waterlogging (Figure 7). This also agrees with the known depressing effect of hypoxia or anoxia on sugar import by sink organs, while source leaves and stems show little change in export and transfer rate, respectively (see Introduction and the review in Geiger & Sovonick, 1975, [43]).

In principle, the effect of waterlogging on phloem unloading by roots must also alter metabolites other than sugars. Here, we found that phloem exudates contained amino acids (with glutamate being the most represented) and organic acids (mostly citrate and malate). The general increase in amino acids under waterlogging (significant for alanine, proline, and glycine) not only reflects the lower consumption by roots, but also likely, the higher production by leaves (Figure 4). The cause for the lower content in organic acids is presently unclear, since malate and citrate in particular are not significantly less abundant in leaf tissue (Figure 4). Waterlogging has consequences on inorganic anions and cations, with a well-known inhibition of nitrate absorption and translocation to shoots via the xylem. Because nitrate is a major counter-anion that is associated with potassium, it is possible that, under our conditions, waterlogging caused a decrease in cation (K^+^, Ca^2+^, Na^+^) content in phloem sap, and this was compensated for by lower organic acid content. In waterlogged *Eucalyptus*, there is a decrease in both Na^+^ and Ca^2+^ in phloem sap [42].

### 3.3. Conclusions and Perspectives

Taken as a whole, our results show that roots and leaves have distinct metabolic responses, not only driven by the effect of oxygen shortage (in roots), but also by the side effect of root hypoxia on sap homeostasis. In particular, our metabolomics data suggest that the decline in unloading efficiency in roots alters sugar translocation, and this exerts a feedback on leaf metabolism so as to redirect sugars to other metabolic pathways. Of course, our present data are limited by the present technology and further work would be needed to provide a full characterization of phloem and xylem composition and flow rate. This would be useful to better appreciate the impact of waterlogging on metabolite circulation. For example, the enhancement of polyamine metabolism in roots and its repression in shoots could be associated with a change in polyamine circulation in both xylem and phloem saps. The exchange of metabolites between roots and other compartments is important, because it could reflect a strategy to export redox power when oxygen limitation that is caused by waterlogging impedes NADH reoxidation in roots. Solving metabolite circulation would require not only metabolomics analyses, but also multiple isotopic tracing, as well as proteomics analyses to identify key enzymes that are up-regulated by waterlogging in an organ-specific manner. This will be addressed in a subsequent study.

## 4. Materials and Methods

### 4.1. Plant Material and Growing Conditions

Barrel clover *Medicago truncatula* (line A17) were grown in pots on neutral substrate and watered each other day with ½ Murashige and Skoog (MS) nutrient solution. The plants were randomly distributed in the growth chamber. Growth conditions were: constant temperature 22 ± 0.2 °C, relative humidity of 70 ± 2 %, long days (16:8 hours light/dark). Three weeks after germination, young plants were subdivided into a set of control plants and one set of plants subjected to waterlogging. Waterlogging was done by filling pots with the nutrient solution. The nutrient solution was replaced every 72 h (with degassed solution). The establishment of hypoxia was checked by measuring dissolved O_2_ content in the pots while using an electrochemical oxygen meter (GMH 3630, Greisinger, Regenstauf, Germany). The control plants were watered with the 1/2 MS nutrient solution. Plants were harvested at the onset of waterlogging treatment (time 0) and after seven and 21 days. 

### 4.2. Biomass and C and N Elemental Content 

At each sampling time, six control and waterlogged plants were harvested. The roots and shoots were collected separately, weighted, frozen in liquid nitrogen, and then stored at −80 °C before further experiments. The plant material was then freeze-dried and grounded in order to obtain a homogeneous fine powder. A sub-sample of 4 mg was used to determine the total nitrogen and carbon content using an elemental analyzer (Thermoquest FlashEA1112®, Thermo Fisher Scientific, Waltham, MA, USA).

### 4.3. Gas Exchange Measurements

The measurement of net photosynthetic assimilation, stomatal conductance, and respiration rates was carried out on four intact plants while using a gas exchange open system Li-Cor 6400 *xt* with the 2 cm^2^ fluorescence chamber (Li-Cor, Austin, TX, USA). Net photosynthesis and stomatal conductance were measured under typical conditions (380 μmol mol^−1^ CO_2_, 21% O_2_, 22 °C, 400 μmol·m^−2^s^−1^ PAR, 10% blue). Dark respiration was measured under the same gaseous conditions after 30 min. dark acclimation. 

### 4.4. Determination of Starch and Soluble Protein Concentrations 

Soluble proteins were extracted from 50 mg frozen plant tissue. Samples were ground to a fine powder, extracted with 2 mL of 100 mM sodium phosphate buffer (pH 7.4) at 4 °C, vortexed three times for 30 s, and then centrifuged at 10,000 g for 20 min. at 4 °C, and the supernatant was collected. Soluble proteins were analyzed in the supernatant with a commercially available kit (Coomassie Protein assay reagent; Bio-Rad, Les Ulis, France) while using bovine serum albumin as a standard. Starch was extracted from 15 mg frozen-dried (lyophilized) plant tissue ground to a fine powder. The samples were depigmented with 1 mL of 80% ethanol and then centrifuged at 10,000 g for 20 min. at 4 °C. Starch was extracted from the pellet by adding 0.2 U of α-amyloglucosidase (Sigma Chemical Co., St. Louis, MO, USA) and 40 U of α-amylase (Sigma Chemical Co., St. Louis, USA) in 20 mM sodium acetate buffer (pH 5.1). Starch was quantified in glucose equivalents determined spectrophotometrically (hexokinase-glucose 6-phosphate dehydrogenase assay).

### 4.5. Phloem Sap Exudation

Shoot phloem exudates were collected over a period of 2 h. The shoots were sectioned at the collar, recut in a Petri dish filled with exudation buffer (5 mM EDTA, pH 6.0), and then immersed in 500 µL of the same buffer. To avoid transpiration, the shoots were placed in dark under very high hygrometry (100% RH). Three biological replicates were used for each point and each nutrition treatment. The exudates were stored at −80 °C until metabolic analysis.

### 4.6. Metabolomics Analyses

Gas chromatography coupled to mass spectrometry (GC-MS) analyses were carried out as in Cui et al., 2019 [26,27]. We used 80% methanol extracts that were derivatized with methoxamine and N-methyl-N-(trimethylsilyl)trifluoroacetamide (MSTFA) in pyridine. Ribitol was used as an internal standard. Two batches of samples were used for metabolomics, which corresponded to two sets of plant cultivation. The use of two batches for metabolomics was decided to ensure that statistical analysis of metabolomics data (with many variables) account for intra-group variability and a decrease the likelihood of false discoveries upon multiple univariate analysis.

### 4.7. Statistics

Physiological variables (photosynthesis, respiration, etc.; Figure 1, Figure 2 and Figure 3) were examined using univariate analysis (one-way or two-way ANOVA, followed by Tukey post-hoc test, and pair-wise comparison with Welch tests, as specified in figure legends). The number of replicates (3 to 5) is indicated in figure legends. 

Metabolomics data were analyzed using both univariate and multivariate statistics. Supervised multivariate analysis was performed with orthogonal projection on latent structure (O2PLS) with Simca 13 (Umetrics, Umea, Sweden), using waterlogging and developmental time as predicted Y variables, and metabolites (UV-scaled) as predicting X variables. We first verified the absence of statistical outliers with principal component analysis (PCA, also done using Simca 13, with UV-scaled metabolite contents as variables), where no data point was outside the 99% confidence Hotelling region. The performance of the multivariate model was assessed while using the determination coefficient R^2^ and the predictive power was quantified by the cross-validated determination coefficient, Q^2^. The significance of the statistical O2PLS model was tested using a χ^2^ comparison with a random model (average ± random error), and the associated *p*-value (*P*_CV-ANOVA_) is reported. The best marker metabolites were visualized with volcano plots, where the logarithm of the *p*-value that was obtained in univariate analysis (two-way ANOVA) was plotted against the rescaled loading (p_corr_) obtained in O2PLS. In such a representation, the best biomarkers have both maximal –log(*P*) and p_corr_ values.

### 4.8. Mass-Balance Calculation of Phloem Loading and Imbalance

Carbon exchange between shoots and roots via the phloem is simplified using a three-compartment model (Figure S2a), where only sucrose is considered. By mass-balance, assimilated carbon is partitioned to net starch synthesis (positive when starch is synthesized; negative when it is degraded), respiration, growth, and export (loading). Similarly, root imported carbon (unloading) is partitioned to respiration, growth, and net starch synthesis. Growth was calculated using the biomass increment (Figure S1) and expressed in sucrose equivalents using %C (Figure 2). Respiration was estimated using the respiration rate that was measured in leaves (Figure 1), converted to a dry mass basis, and rescaled to the total biomass of the organ considered. Starch content was measured directly (Figure 2). When rates are expressed in µmol sucrose shoot^−1^ d^−1^, the mass-balance applied to the shoot sucrose total pool (*S_s_*) is so that:

(1) $$\frac{dS_{s}}{dt}=A-R_{s}-G_{s}-\sigma_{s}-C$$

Because the total sucrose pool equals the average concentration (*ω_s_*, µmoles per g DW) times biomass (*B_s_*, in g DW), we have *S_s_* = *B_s_ω_s_*. Assuming that variations in *ω_s_* are small as compared to biomass increase (i.e. the order of magnitude of sucrose concentration does not change dramatically), we have:

(2) $$\frac{dS_{s}}{dt}\approx \omega_{s}\frac{dB_{s}}{dt}=\omega_{s}\xi G_{s}$$

where *ξ* is the conversion factor of growth (*G_s_*) from dry mass to µmoles sucrose (0.00036 g DW µmol^−1^ sucrose). Combining (1) and (2) gives:

(3) $$C=A-R_{s}-G_{s}\cdot \left(1+\xi \omega_{s}\right)-\sigma_{s}$$

Similarly, for roots, we obtain:

(4) $$D=R_{r}+G_{r}\cdot \left(1+\xi \omega_{r}\right)+\sigma_{r}$$

where *ω_r_* is sucrose concentration in roots.

The phloem imbalance (denoted as *i*) is then given by the difference between loading and unloading, *i* = *C* − *D*. By definition, this imbalance represents the incremental change in total phloem sucrose pool (*S_p_*, in µmol phloem sucrose plant^−1^) with time, which is:

(5) $$\frac{dS_{p}}{dt}=i=C-D$$

When this difference is positive, *S_p_* increases. This is the general case, since the plant size increases and so must be total phloem volume. Nevertheless, because *S_p_* = *V_p_*∙*ω_p_* (where *V_p_* is total phloem volume and *ω_p_* phloem sucrose concentration) and *V_p_* is not readily accessible, *S_p_* cannot be converted into a concentration. Computations indicate that *S_p_* is within 1 and 10 µmol plant^−1^. For example, if we assume that *V_p_* is about 2.5% of total plant volume, it means an average concentration *ω_p_* of 40 mM (14 mg mL^−1^) in phloem sap, which is a realistic value. In Figure 7, the imbalance was also expressed in percentage (denoted as *p*) of sucrose loading, as follows:

(6) $$p=\frac{i}{C}$$

## Supplementary Materials

The following are available online at https://www.mdpi.com/2223-7747/9/10/1373/s1, Figure S1: Plant biomass and relative water content, Figure S2: Model used to estimate the rate of phloem loading.
