# Supplementary material for: Metabolic Responses to Waterlogging Differ between Roots and Shoots and Reflect Phloem Transport Alteration in Medicago truncatula
Source: Plants (Basel). 2020 Oct 15;9(10):1373. doi: 10.3390/plants9101373 (PMC7650564; doi:10.3390/plants9101373)
Supplement: Supplementary file 1 [file plants-09-01373-s001.pdf]

Supplementary material

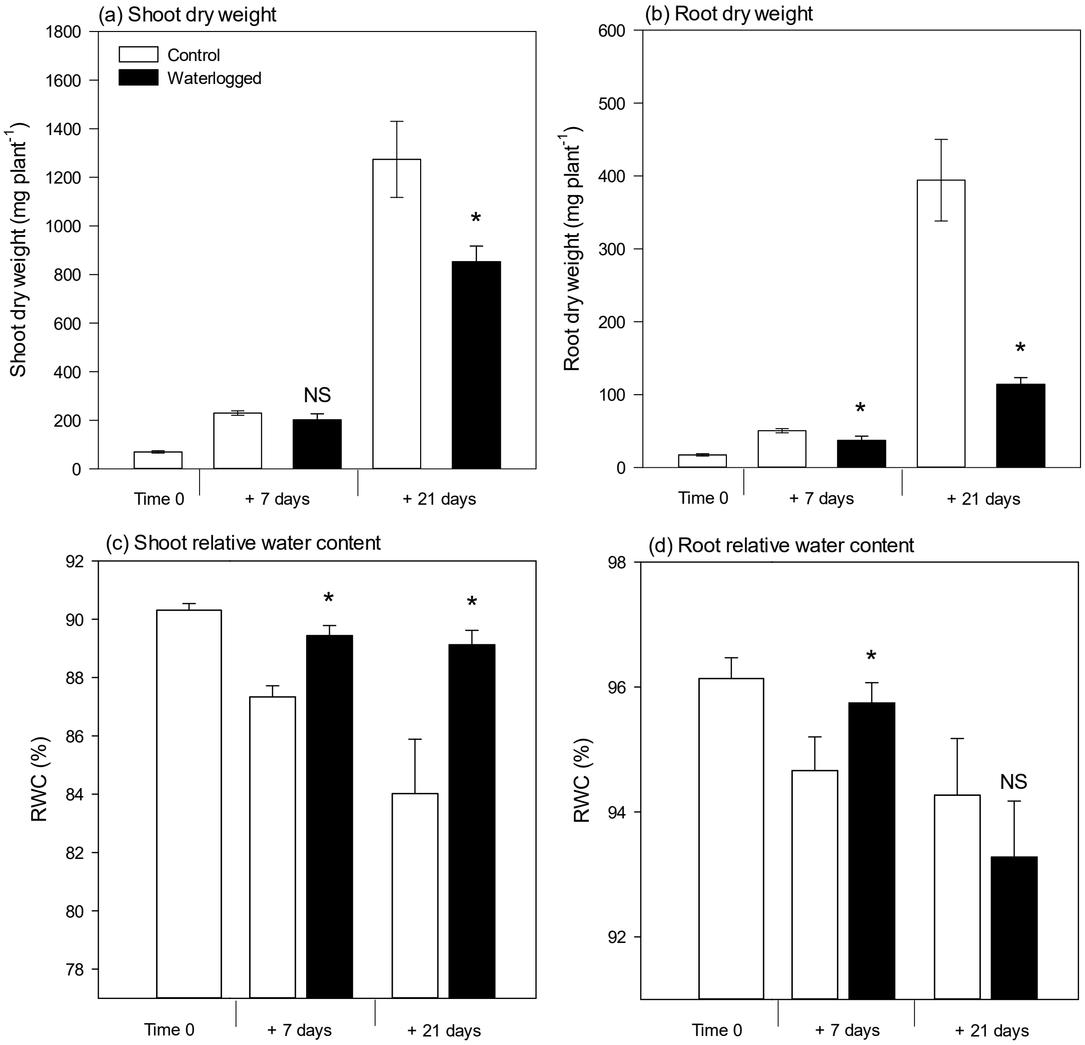

Figure 1. Plant biomass (**a**,**b**) and relative water content (**c**,**d**) seven and 21 days after the beginning of waterlogging. The zero-time point was just before the onset of waterlogging. Non-waterlogged plants (control plants) are shown with open bars, waterlogged plants with closed bars. Data are mean ± SE (n = 6). Asterisks stand for significance (p < 0.05, Tukey test) between control and waterlogging. NS, non significant.

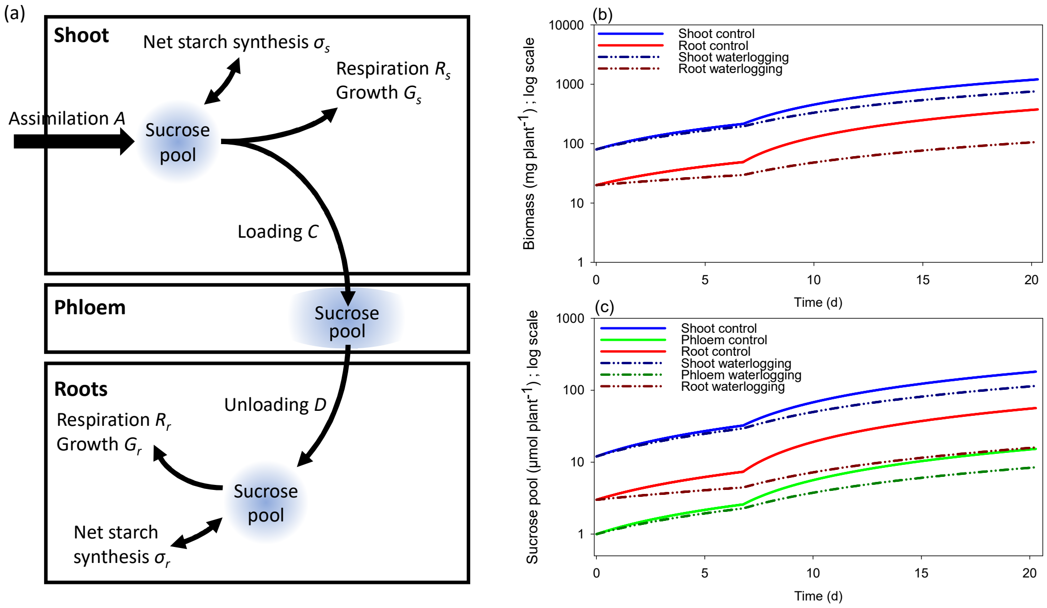

Figure S2. Model used to estimate the rate of phloem loading. (**a**) scheme depicting sucrose pools considered here. (**b**) modelled time course of biomass (shoots and roots). (c) modelled sucrose pools (total sucrose in the compartment of interest, in µmol plant^{-1}). Total sucrose pools increase with time due to the general increase in plant size. See Notes S1 for further details on modelling. Note the use of the $\log_{10}$ scale in (**b**) and (**c**).

Notes S1. Description of the mass-balance calculation of phloem loading and imbalance

Carbon exchange between shoots and roots via the phloem is simplified using a three-compartment model (Fig. S2.a) where only sucrose is considered. By mass-balance, assimilated carbon is partitioned to net starch synthesis (positive when starch is synthesized; negative when it is degraded), respiration, growth and export (loading). Similarly, root imported carbon (unloading) is partitioned to respiration, growth and net starch synthesis. Growth was calculated using the biomass increment (Fig. S1) and expressed in sucrose equivalents using %C (Fig. 2). Respiration was estimated using the respiration rate measured in leaves (Fig. 1), converted to a dry mass basis and rescaled to the total biomass of the organ considered. Starch content was measured directly (Fig. 2). When rates are expressed in µmol sucrose shoot^{-1} d^{-1}, the mass-balance applied to the shoot sucrose total pool (S_s) is so that:

$$\frac{dS_s}{dt} = A - R_s - G_s - \sigma_s - C \tag{1}$$

Since the total sucrose pool equals the average concentration (ω_s, µmoles per g DW) times biomass (B_s, in g DW), we have $S_s = B_s\omega_s$. Assuming that variations in ω_s are small to compared to biomass increase (i.e. the order of magnitude of sucrose concentration does not change dramatically), we have:

$$\frac{dS_s}{dt} \approx \omega_s \frac{dB_s}{dt} = \omega_s \xi G_s \tag{2}$$

where ξ is the conversion factor of growth (G_s) from dry mass to µmoles sucrose (0.00036 g DW µmol^{-1} sucrose). Combining (1) and (2) gives:

$$C = A - R_s - G_s \cdot \left(1 + \xi\omega_s\right) - \sigma_s \tag{3}$$

Similarly, for roots, we obtain:

$$D = R_r + G_r \cdot \left(1 + \xi\omega_r\right) + \sigma_r \qquad (4)$$

where ω_r is sucrose concentration in roots.

The phloem imbalance (denoted as i) is then given by the difference between loading and unloading, $i = C - D$. By definition, this imbalance represents the incremental change in total phloem sucrose pool (S_p, in µmol phloem sucrose plant^{-1}) with time, that is:

$$\frac{dS_p}{dt} = i = C - D \qquad (5)$$

When this difference is positive, S_p increases. This is the general case since the plant size increases and so must be total phloem volume. Nevertheless, since $S_p = V_p \cdot \omega_p$ (where V_p is total phloem volume and ω_p phloem sucrose concentration) and V_p is not readily accessible, S_p cannot be converted into a concentration. Computations indicate that S_p is within 1 and 10 µmol plant^{-1}. For example, if we assume that V_p is about 2.5% of total plant volume, it means an average concentration ω_p of 40 mM (14 mg mL^{-1}) in phloem sap, which is a realistic value. In Fig. 7, the imbalance was also expressed in percentage (denoted as p) of sucrose loading as follows:

$$p = \frac{i}{C} \qquad (6)$$
